# CRISPR screening of E3 ubiquitin ligases reveals Ring Finger Protein 185 as a novel tumor suppressor in glioblastoma repressed by promoter hypermethylation and miR-587

**DOI:** 10.1186/s12967-022-03284-z

**Published:** 2022-02-19

**Authors:** Kun Lin, Shang-Hang Shen, Feng Lu, Pengfeng Zheng, Shizhong Wu, Jingwei Liao, Xiaohang Jiang, Guangming Zeng, De Wei

**Affiliations:** 1 Department of Neurosurgery, Fujian Provincial Hospital South Branch, 516 Jinrong South Road, Fuzhou, 350001 China; 2 Department of Neurosurgery, Shengli Clinical Medical College of Fujian Medical University, 516 Jinrong South Road, Fuzhou, 350001 China; 3 Department of Neurosurgery, The First Affiliated Hospital of Xiamen University, Medical College of Xiamen University, Xiamen, 361003 China; 4 Department of Neurosurgery, Fujian Provincial Hospital, 134 East Street, Fuzhou, 350001 China; 5 Department of Neurosurgery, Shengli Clinical Medical College of Fujian Medical University, 134 East Street, Fuzhou, 350001 China

**Keywords:** Glioblastoma, CRISPR screening, E3 ubiquitin ligases, Ring Finger Protein 185, Promoter hypermethylation, miR-587

## Abstract

Glioblastoma (GBM) is the most common and aggressive malignant primary brain tumor. E3 ligases play important functions in glioma pathogenesis. CRISPR system offers a powerful platform for genome manipulation, while the screen of E3 ligases in GBM still remains to be explored. Here, we first constructed an E3 ligase small guide RNA (sgRNAs) library for glioma cells growth screening. After four passages, 299 significantly enriched or lost genes (SELGs) were compared with the initial state. Then the clinical significance of SELGs were validated and analyzed with TCGA glioblastoma and CGGA datasets. As RNF185 showed lost signal, decreased expression and favorable prognostic significance, we chose RNF185 for functional analysis. In vitro overexpressed cellular phenotype showed that RNF185 was a tumor suppressor in two glioma cell lines. Finally, the molecular mechanism of decreased RNF185 expression was investigated and increased miR-587 expression and DNA hypermethylation was evaluated. This study would provide a link between the molecular basis and glioblastoma pathogenesis, and a novel perspective for glioblastoma treatment.

**Supplementary Information:**

The online version contains supplementary material available at 10.1186/s12967-022-03284-z.

## Introduction

Glioblastoma (GBM) is the most common and aggressive malignant primary brain tumor, categorized as grade IV diffuse glioma by the World Health Organization (WHO) [1–4]. As in other cancers, glioma progression is accompanied by abnormal molecular changes, such as chromosomal instability [5], IDH1/2 mutation [6], EGFR amplification [7] and 1p/19q deletions [8]. Even though current advances in surgery, chemotherapy and immunotherapy [9–11], the prognosis of GBM patients still remains poor [12]. Therefore, there is an urgent need to explore the exact molecular mechanisms of glioma progression and develop new and effective treatment strategies to improve patient prognosis.

The ubiquitin–proteasome system regulates many cellular processes, including cell cycle, differentiation, DNA repair, and the immune response in cancer [13]. Accumulating evidence shows that E3 ligases play important functions in glioma pathogenesis, progression, therapy response and prognostic marker [14]. For example, Cullin-7 (CUL7) plays a significant role in promoting gliomagenesis via NF-κB activation [15]. FBXO16, a component of SCF E3 ubiquitin ligase complex has been proved to mediateβ-catenin degradation, and its attenuation could activate Wnt signaling and promote glioblastoma [16]. Additionally, the ubiquitin–proteasome system and E3 ligases have been proposed as therapeutic targets in cancers, including glioma [17, 18]. Thereby, reveals the role of E3 ligases in glioblastoma would provide novel targets for the therapy of glioma.

Clustered regularly interspaced short palindromic repeats (CRISPR) system offers a powerful platform for genome manipulation, including protein-coding genes, noncoding RNAs and regulatory elements [19, 20]. The development of CRISPR screen enables high-throughput interrogation of gene functions in diverse tumor biologies, such as tumor growth, metastasis, synthetic lethal interactions, therapeutic resistance and immunotherapy response [21–24]. For instance, genome wide CRISPR screening was applied and revealed E3 ubiquitin ligase Rnf20 as a negative regulator of Foxp3 in regulatory T (Treg) cells [25]. In glioblastoma, CRISPR screen has been applied to identify functional suppressors [26], therapeutic targets [27–29], membrane targets for improving immunotherapy [30] as well as mechanisms of temozolomide sensitivity [31, 32].

Here, we first constructed an E3 ligase small guide RNA (sgRNAs) library for glioma cells survival screening. After 4 passages, significantly enriched or lost genes were compared with the initial state. Then the clinical significance of significantly enriched or lost genes were validated and analyzed with TCGA glioblastoma and CGGA datasets. As one E3 ligase RNF185 showed lost signal, decreased expression and favorable prognostic significance, we chose RNF185 for functional analysis. In vitro overexpressed cellular phenotype showed that RNF185 was a tumor suppressor in two glioma cell lines. Finally, the molecular mechanism of decreased RNF185 expression was investigated and increased miRNAs expression and DNA methylation was evaluated. This study would provide a link between the molecular basis and glioblastoma pathogenesis, and a novel perspective for glioblastoma treatment.

## Material and methods

### CRSIRP screening library constriction

SgRNAs of 557 E3 ubiquitin ligases were designed, and gene knockdown efficiency was validated with qRT-PCR examinations. The ubiquitin ligase gene list and validated sgRNA sequences were shown in Additional file 1: Table S1.

### Identification of differential genes in CRSIRP screening

Based on the amplicons sequencing, pooled sequencing reads were compared with human genome HG38. The reads of E3 ligase genes in the fourth passage were compared with the passage 0. By using reads fold change ≥ 2 or ≤ 0.5 and p value < 0.05, differential genes were screened.

### Clinical significance analysis of genes in TCGA GBM and CGGA datasets

Class-three gene expression of GDC TCGA Glioblastoma (GBM) dataset was downloaded from UCSC Xena website (https://xena.ucsc.edu/), and applied for subsequent survival analysis. Log-Rank test of patients with higher and lower gene expression was conducted, and Kaplan–Meier survival plot was shown. For Chinese Glioma Genome Atlas (CGGA) analysis, gene expression and prognostic significance in WHO II–III grade glioma samples, the relationship of gene methylation patterns and parameter of brain cancer etiology (including Histology type, WHO grade, IDH mutation status and 1p/19q deletion) was conducted with the online tool.

### Cell culture and transfection

Glioblastoma cells lines U87, U251 and DBTRG were purchased from the Cell Bank Type Culture Collection of the Chinese Academy of Sciences (Shanghai, China). Cells were cultured in DMEM (Gibco, Carlsbad, CA, USA) supplemented with 10% of fetal bovine serum (Gibco) and 1% of penicillin–streptomycin at 37 °C, 5% CO_2_ humidified atmosphere. For cell transfection, the cells were cultured in 6 well plates, when the cell reached 75% confluence. The negative control, pcDNA3.1-RNF185, negative control, and miR-587 inhibitors were synthesized by Shanghai Gene Pharma Co., Ltd (Shanghai, China). The negative control, pcDNA3.1-RNF185 were transfected into glioblastoma cells using Lipofectamine 2000 transfection reagent (Invitrogen, USA) according to manufacturer’s instructions. Negative control or miR-587 inhibitors at concentration of 5 nM, 10 nM, 20 nM, 40 nM were transfected into glioblastoma cells using lipofectamine 2000 transfection reagent according to manufacturer’s instructions. The overexpression and knockdown efficiency were validated with qRT-PCR. GAPDH and U6 were used as the house keeping gene. Primers for RNF185, miR-587, GAPDH and U6 are as follows: RNF185: F-5′-GTGTTTACATCAGTGGTTGGAGA-3′; R-5′-GTGCTGCCCCTTCCATAGAG-3′;

GAPDH: F-5′-GGAGCGAGATCCCTCCAAAAT-3′; R-5′-GGCTGTTGTCATACTTCTCATGG-3′;

miR-587: F-5′-CCAGGCAAGAGAGAGTTGCTG-3′; R-5′-AGTCACAGGTGCAGACACATT-3′;

U6: F-5′-CTCGCTTCGGCAGCACA′; R-5′-AACGCTTCACGAATTTGCGT-3′. Anti-miR-587 miScript miRNA inhibitor was purchased from Qiagen Inc. (Valencia, CA, USA).

### Cell counting kit-8 (CCK-8)

U251 and DBTRG cells were seeded into 96-well plates (2 × 10^4^ cells/well) and cultured for 12 h. After washing, U251 and DBTRG cells were incubated with 10% CCK-8 (Dojindo Molecular Technologies, Inc., Minato-ku, Tokyo, Japan) and optical density measured using a xMark Microporous Plate Absorption Spectrophotometer (Bio-Rad Laboratories, Inc., Hercules, CA, USA).

### Cell apoptosis assay

The apoptotic rate of U251 and DBTRG cells was detected using an Annexin V, 633 Apoptosis Detection Kit (Dojindo Molecular Technologies), following the kit instructions. U251 and DBTRG cells were seeded into 6-well plates (5 × 10^5^ cells/well) and cultured for 12 h. Next, U251 and DBTRG cells were incubated with Annexin V, followed by propidium iodide (PI) buffer for 15 min at 25 °C in a dark room. Subsequently, apoptotic cells were quantified using a NovoCyte 1040 flow cytometer (ACEA Biosciences, Inc., Zhejiang, China).

### Western blot

Cells were lysed in a mixed buffer that contained RIPA, NaF, and PMSF. Protein concentrations were analyzed using a BCA protein assay kit (Tiangen Biotech Co., Ltd., Beijing, China). Protein was resolved by 10% SDS-PAGE and transferred to PVDF membranes (Millipore, Bedford, MA, USA). Membranes were incubated overnight with the indicated primary antibodies at 4 °C and then incubated with appropriate secondary antibodies for 2 h at room temperature. Protein bands were detected by ImageQuant LAS4000 (General Electric Company, Boston, MA, USA) and quantified by ImageJ software. GAPDH was detected as a loading control. Primary antibodies used in the study were listed as the following: TNFR1 (Proteintech, Cat No. 60192-1-AP), BAD (Proteintech, Cat No. 10435-1-AP), FAS (Proteintech, Cat No. 13098-1-AP), Cleaved Caspase3 (Proteintech, Cat No. 66470-2-Ig), and GAPDH (Proteintech, 60004-1-Ig).

### Wound healing assay

The migratory ability was assessed through wound-healing assay. U251 and DBTRG cells (2 × 10^5^) were seeded into a 6-well plate allowed to reach confluence. Then, uniform wounds were scraped using a 200-µl pipette tip across the cell monolayer. Cells were rinsed with phosphate-buffered saline and cultured in the medium. Then, the wound closures were observed after 24 h. The initial gap length (0 h) and the residual gap length (24 h) after wounding were calculated from photomicrographs using an Olympus fluorescence microscope (Olympus).

### Dual luciferase assay

PmirGLO Dual-Luciferase miR Target Expression Vector (Promega) was used to assess the direct binding of miR-587 to RNF185 3′UTR. The wild-type reporter construct pmirGLO-RNF185-WT or the mutant reporter construct (pmirGLO-RNF185-MUT) with miR-587 inhibitors were co-transfected in 293T cells. After transfection for 24 h, firefly luciferase levels were measured using a Dual-Luciferase Reporter Assay System (Promega, Wisconsin) and normalized to Renilla luciferase activity. Each experiment was repeated at least three times.

### Trans-well assay

For the trans-well assay, Matrigel were seeded into the upper chamber. The 3 × 10^5^ (without Matrigel) or 5 × 10^5^ cells (with Matrigel) in serum‐free medium were seeded into the upper chamber. The lower chamber was filled with medium supplemented with 10% FBS as a chemo-attractant. After 48 h of incubation, the cells on the upper surface of the filter were removed with a cotton swab, and cells that invaded through the filter or Matrigel sticked to the lower surface of the filter, were fixed and stained with 0.5% crystal violet, and counted under a light microscope.

### Statistical analysis

All statistical analyses were performed using Graphpad v 8.0 software. Each experiment was performed in triplicates, and the data were shown as the mean ± SD, unless otherwise stated. Kaplan–Meier analysis with log-rank test was used for survival analysis. Student’s t-test was used to compare the mean values. Pearson chi-square test was used to analyze the association between RNF185 expression and miRNAs. P value < 0.05 was considered to be statistically significant.

## Results

### E3 ubiquitin ligases library CRISPR screening reveals glioma cell growth regulators

**Fig. 1:**
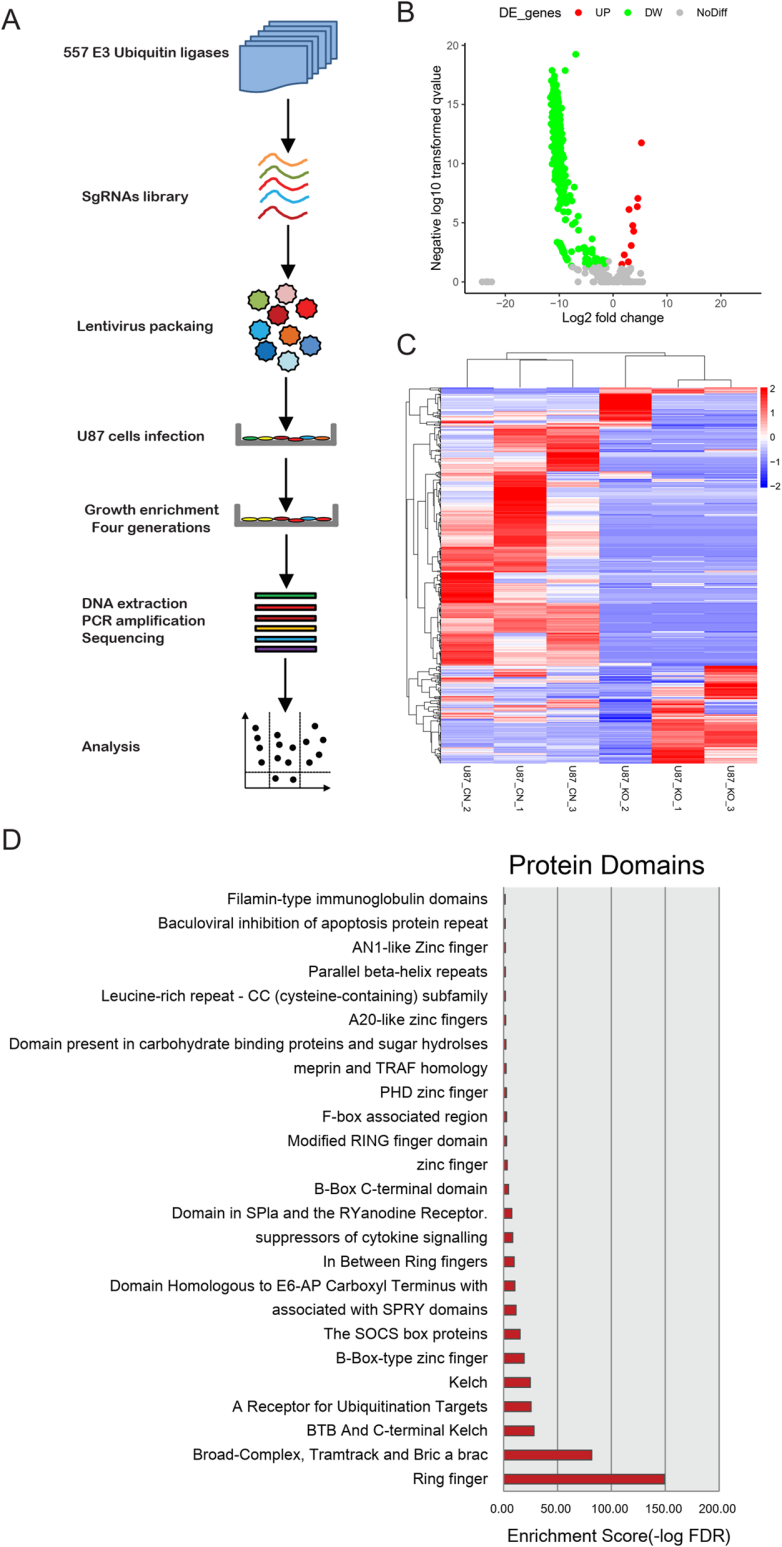
CRISPR screening of E3 ligases reveals significantly changed genes (SCGs) in glioma cell line U87. **A** The workflow chat of CRISPR screening assays of E3 ligases in glioma U87 cells; **B** volcano plot showing the significantly enriched and lost genes; enriched genes were shown with red dots, and lost genes were shown with green dots; **C** reads counts in the first and 4th generation cells; **D** protein domain analysis of enriched and lost genes

To explore the function of E3 ubiquitin ligases on tumor growth phenotype of glioma, a sgRNAs library of 557 E3 ligase genes were constructed, and followed by lentivirus packaging. Then glioma cancer cell U87 was used as a representative cell line for the subsequent cell growth screening. After the cell passage for 4 generations, cells were collected, and the DNA amplicons were applied for sequencing. The identified gene reads in 4^th^ generation were compared with the initial gene reads, and cell with enriched and lost reads were statically compared (Fig. 1A). By using reads number cutoff of ≥ 2 or ≤ 0.5 and p-value < 0.05, all 299 (9 enriched and 290 lost) significantly enriched or lost genes (SELGs) were acquired (Fig. 1B). The reads of enriched or lost genes in all 3 replicates were shown in Fig. 1C. At last, the domain of significant enriched proteins was analyzed, and as shown in Fig. 1D, proteins with Ring finger ranked top one, followed by Broad-Complex, BTC and C-termincal Kelch. The sgRNAs sequences of 557 E3 ubiquitin ligases and analyzed results were shown in Additional file 1: Table S1.

### Expression validation and prognostic analysis reveals clinical markers in glioma

**Fig. 2:**
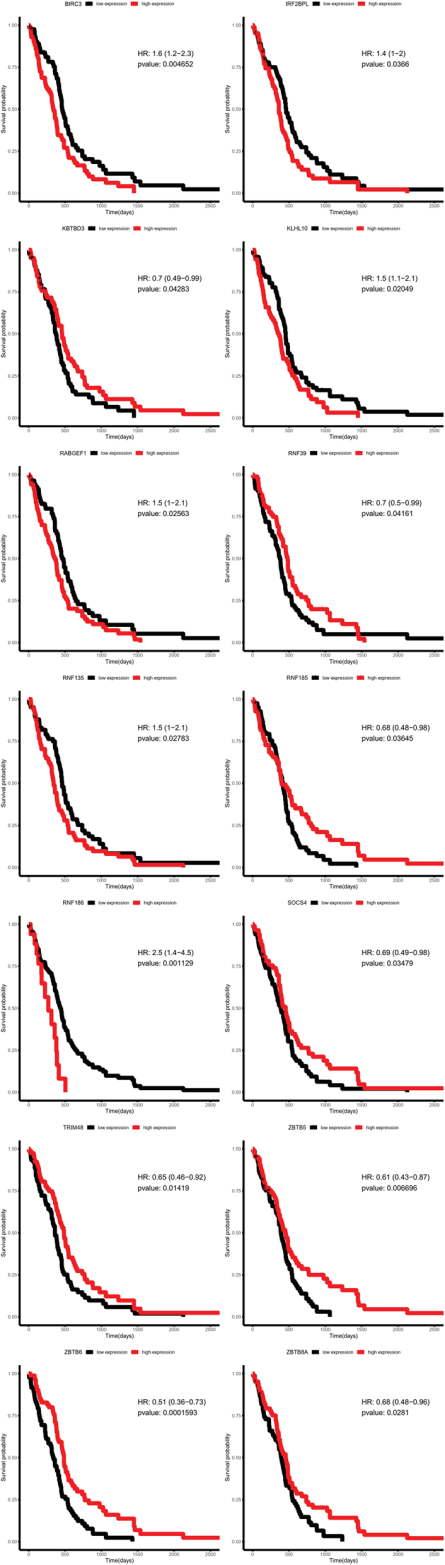
Kaplan–Meier survival plot showing the prognostic performance of 14 genes in TCGA glioblastoma (GBM) datasets

**Fig. 3:**
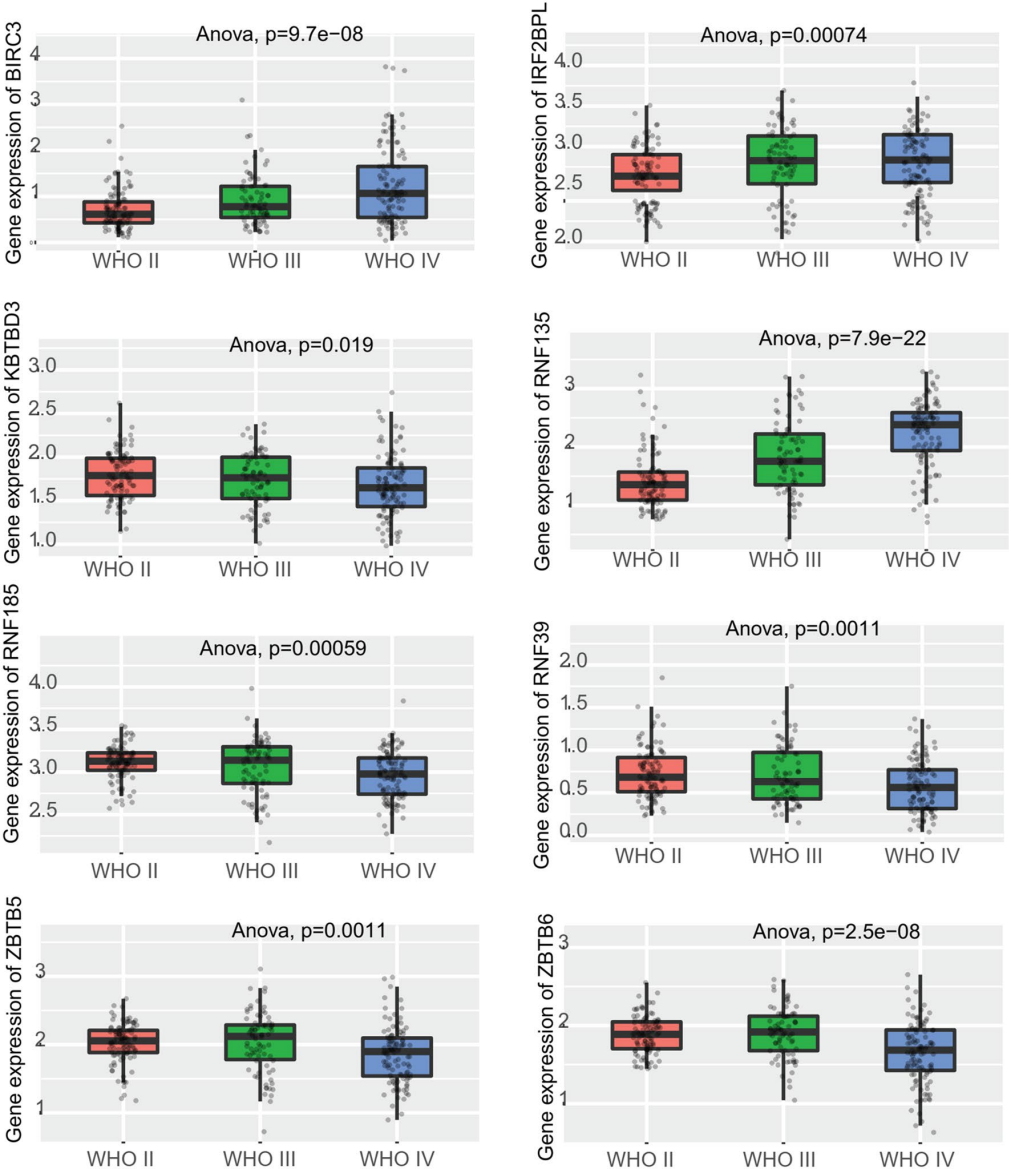
Expression box plot showing the expression of 8 genes in WHO II, III and IV grade glioma samples, in CGGA dataset

**Fig. 4:**
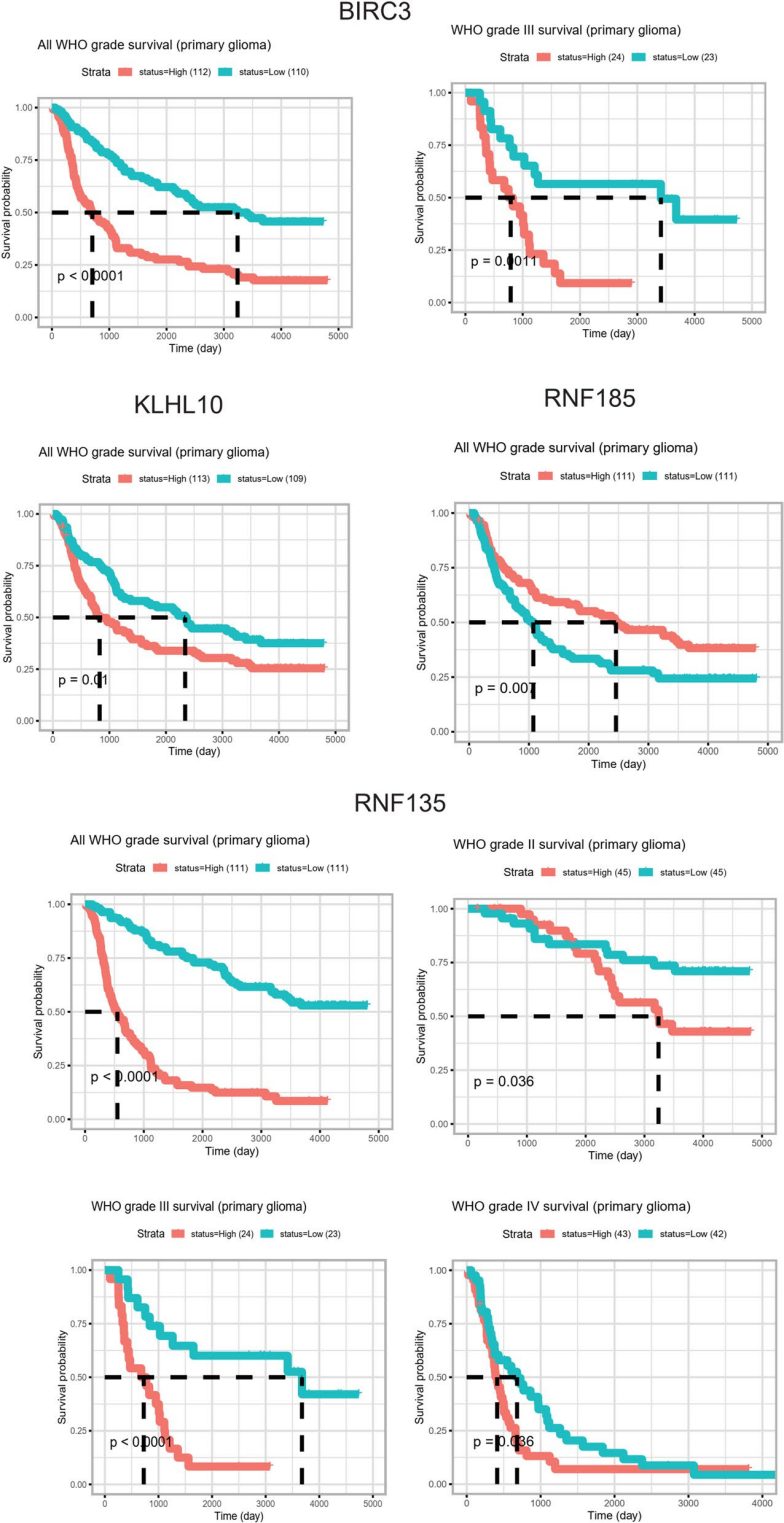
Kaplan–Meier survival plot showing the prognostic performance of 4 genes in CGGA dataset

Then the clinical significance of all SCGs was conducted for further analysis. First, the overall survival analysis was conducted with TCGA glioma multiforme (GBM) dataset. By using median expression of each SCG as cut-off, Log-Rank analysis was applied to calculate the Hazard ratio and p-value. All 14 genes (BIRC3, IRF2BPL, KBTBD3, KLHL10, RABGEF1, RNF39, RNF135, RNF185, RNF186, SOCS4, TRIM48, ZBTB5, ZBTB6 and ZBTB8A) showed p-value < 0.05, and the Kaplan–Meier survival plot was shown in Fig. 2. Then the 14 prognostic genes were conducted for further analysis, with CGGA datasets. First, the expression of these 14 genes was compared in WHO II, III and IV grade. As shown in Fig. 3, 8 genes showed significant deregulation in GBM II-IV grade groups, such as BIRC3, IRF2BPL and RNF135 showed consistent higher expression, and decreased expression was observed of genes KBTBD3, RNF185, RNF39, ZBTB5 and ZBTB6. Finally, the prognostic significance of these 14 genes was further analyzed in CGGA datasets, and four genes showed significance. BIRC3, KLHL10 and RNF135 showed unfavorable biomarker performance in glioma samples, and only RNF185 may serve as a favorable marker (Fig. 4).

### Ring Finger Protein 185 inhibit glioma cancer cell proliferation and migration

**Fig. 5:**
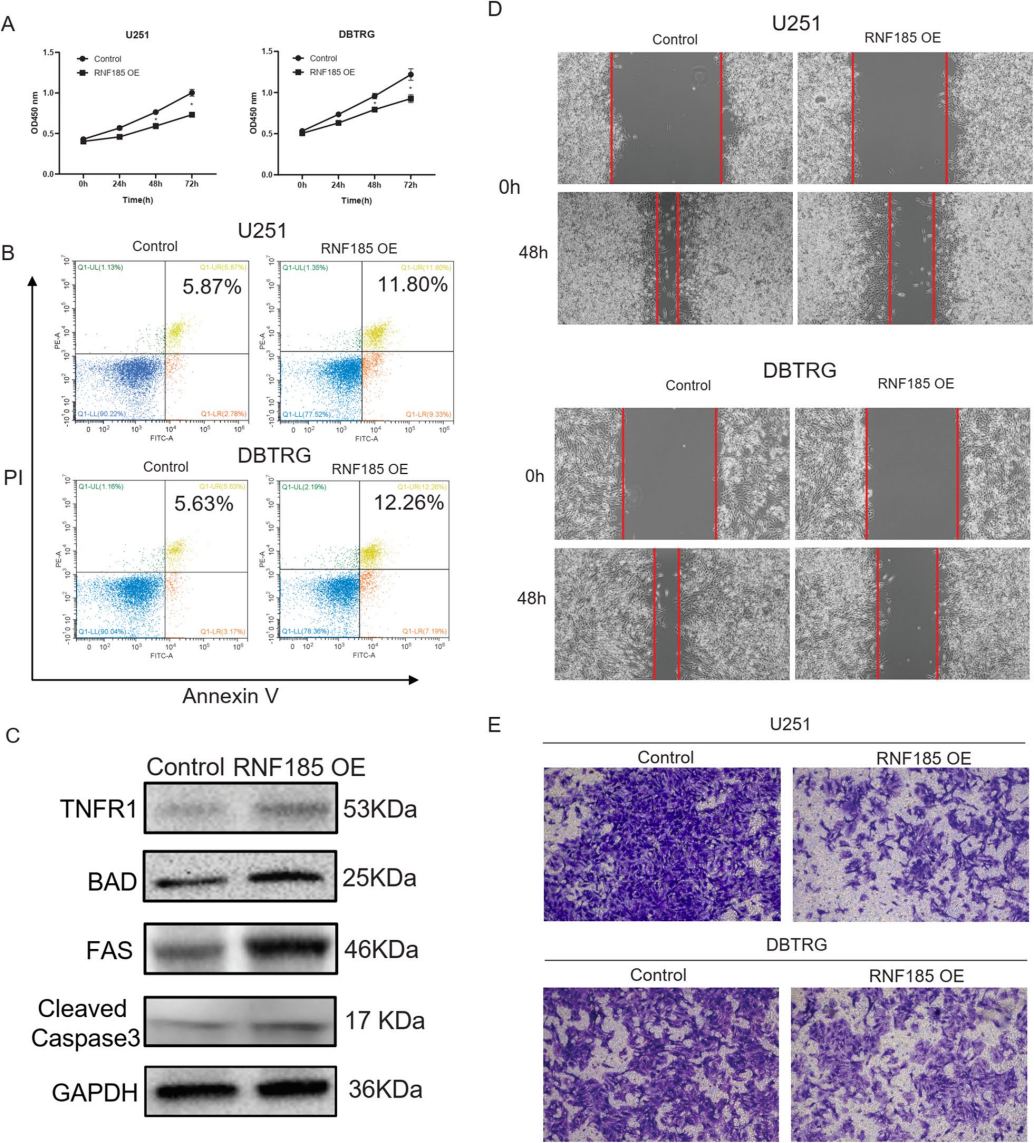
In vitro study of the function of RNF185 overexpression in glioma cells U87 and DBTRG. **A** CCK8 assays showing the function of RNF185 overexpression in glioma cell number; **B** FACS determination of the function of RNF185 overexpression in glioma cell apoptosis rate; **C** wound healing assay studying the function of RNF185 overexpression in glioma cell migration; All experiments were conducted for three replicates; *p < 0.05, **p < 0.01

Of the 3 genes (BIRC3, RNF135 and RNF185) with both differential expression and prognostic significance, BIRC3 [33–35] and RNF135 [36] were all previously reported as oncogenes in glioma. Ring Finger Protein 185 (RNF185) has been shown to regulate cell autophagy [37], innate immune responses [38], osteogenic differentiation [39] and cystic fibrosis [40]. Moreover, RNF185 was proved to promote gastric cancer metastasis [41], while its role in glioma remains to be explored. Interestingly, though the function of RNF185 was shown to promote gastric cancer, our screening results in U87 cells showed RNF185 as a tumor suppressor. So, we further validated the function of RNF185 in other glioma cell lines, U251 and DBTRG, with overexpression strategy. As the CCK8 assay results showed in Fig. 5A, RNF185 overexpressed U251 and DBTRG cells exhibits decreased cell number, suggesting that RNF185 inhibits cell proliferation. Then we examined the cell apoptosis rate, and RNF185 overexpressed U251 and DBTRG cells demonstrated significant higher apoptosis rate, compared with control cell lines (Fig. 5B). Western blotting assay revealed that overexpression of RNF185 significantly increased the protein level of TNFR1, BAD, FAS, and Cleaved Caspase3 (Fig. 5C), indicating the promotion of apoptosis. At last, the migration capability was studied with wound healing assays, and as shown in Fig. 5D, RNF185 overexpression significantly attenuated the migration distance in both cell lines. Transwell assay also found that overexpression of RNF185 inhibited the invasion of U251 and DBTRG cells (Fig. 5E). In summary, the above results proved RNF185 as a tumor suppressor in glioma cell lines.

### Decreased RNF185 was caused by increased miR-587 expression

**Fig. 6:**
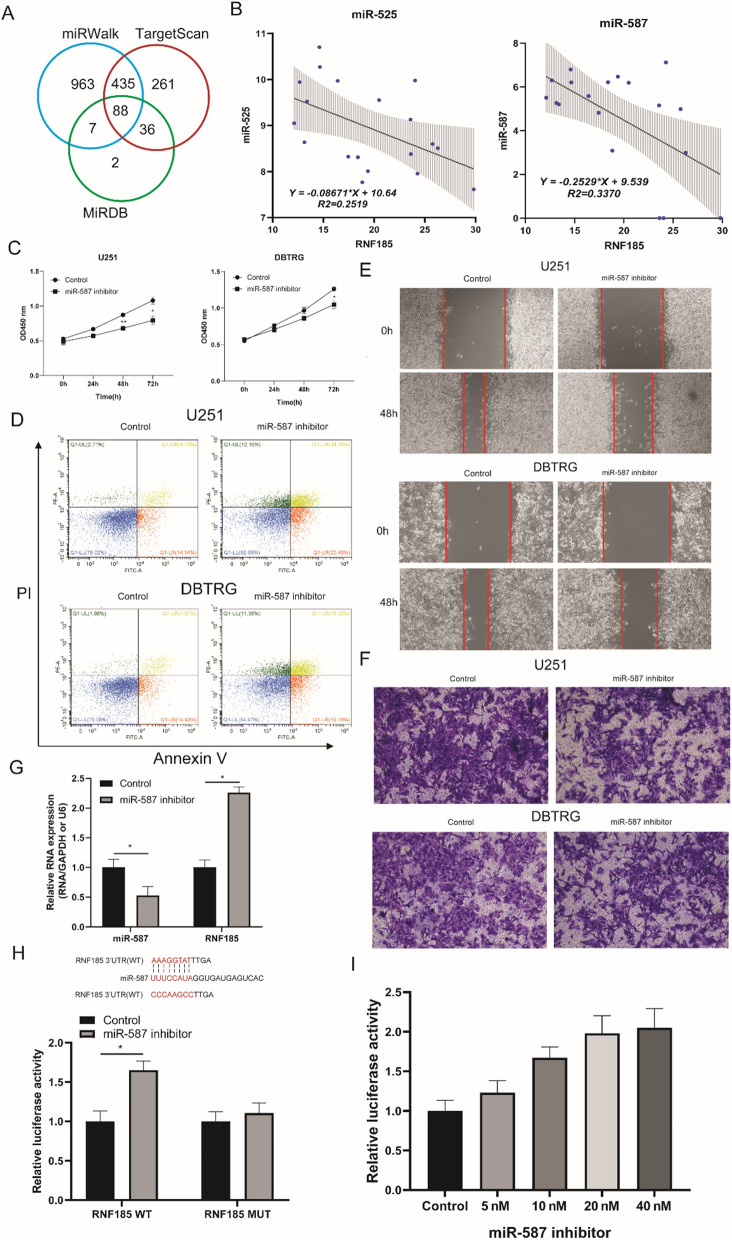
MiR-587 regulates RNF185 expression and promotes glioma cell proliferation and migration. **A** The intersection analysis of predicted miRNAs by three online tools; **B** correlation analysis of RNF185 with miR-525 and miR-587; **C** CCK8 assays showing the cell number of miR-587 inhibitor treated glioma cells; **D** Annexin V/PI staining demonstrates the function of miR-587 inhibitor on glioma cells apoptosis; **E** wound healing assay shows the function of miR-587 inhibitor on glioma cells migration; **F** the expression of RNF185 in miR-587 inhibitor treated glioma cells; **G** dual luciferase assay showing the binding site and regulatory effect of miR-587 on RNF185 mRNA; All experiments were conducted for three replicates; *p < 0.05, **p < 0.01

Next, we intend to explore the reason for the decreased expression of RNF185 in glioma cancer samples. MicroRNAs (miRNAs) have been shown to mediated mRNA degradation and repress protein translation by targeting mRNA 3′UTRs, in post-transcriptional level. In order to explore miRNAs that may regulate RNF185 expression in glioma, we first predicted miRNAs with three online tools: miRWalk, TargetScan and miRDB. By comparing the miRNAs predicted in all three tools, 88 common miRNAs were obtained (Fig. 6A). Then the correlation of these 88 miRNAs with RNF185 expression were analyzed with TCGA glioma multiforme (GBM) dataset, and miR-525 and miR-587 showed significant negative correlation with RNF185 (Fig. 6B). As miR-525 was shown to repress glioma cells proliferation in other studies [42], we chose miR-587 for subsequent analysis, for its oncogenic role in multiple cancer types [43–45] except glioma. So, we first studied the function of miR-587 in glioma cells with CCK8, apoptosis, wound healing assays and transwell assays. As shown in Fig. 6C–F, miR-587 inhibitors phenol-copied the cell proliferation, apoptosis and migration and invasion function of RNF185 overexpressed U251 and DBTRG cells, implying that miR-587 play an oncogenic role in glioma. Next, to explore whether miR-587 regulate RNF185 expression, we examined the expression of RNF185 in miR-587 inhibitor treated glioma cells. As a result, miR-587 inhibitors significantly increased the RNF185 (Fig. 6G). Finally, a dual luciferase assay was conducted to validate the binding of miR-587 on RNF185 3’UTR sequences, by mutating the predicted sites of RNF185 3’UTR sequences. As shown in Fig. 6H, RNF185 wide type (WT) cells showed increased luciferase activity, while no significant changes were observed in mutant (MUT) cells. RNF185 wide type (WT) cells showed increased luciferase activity with dose dependence of miR-587 inhibitor reagent (Fig. 6I). Taken together, elevated miR-587 expression contributes to the decreased expression of RNF185 and promotes glioma proliferation, migration and reduces cell apoptosis.

### Promoter hypermethylation correlated with decreased RNF185 expression

**Fig. 7:**
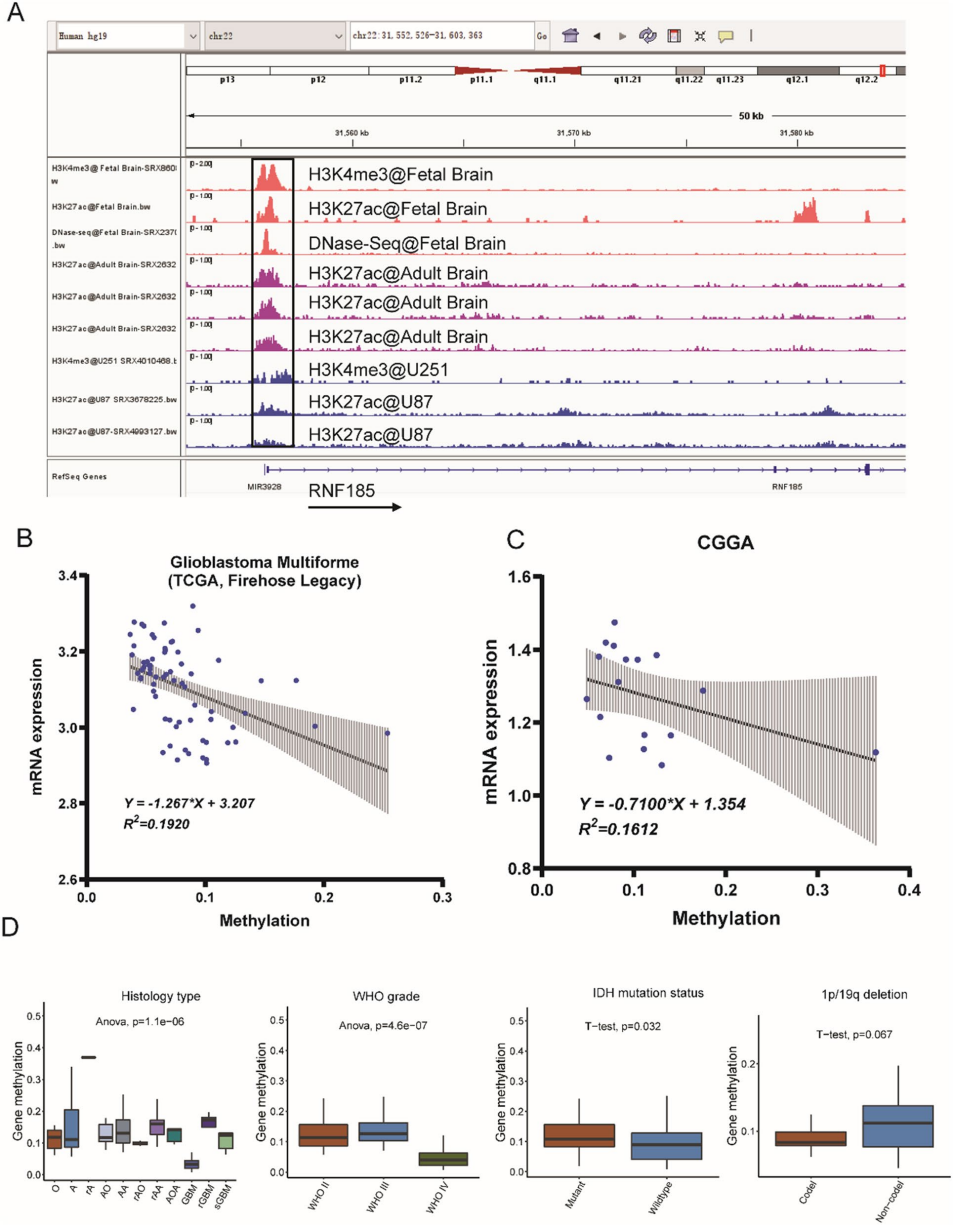
Transcriptional analysis of RNF185 expression signals in glioma. **A** ChIP-Seq binding peaks in fetal brain, adult brain and glioma cells U87 and U251; **B** correlation analysis of DNA methylation level with the RNF185 mRNA expression level in TCGA glioblastoma multiforme dataset; **C** correlation analysis of DNA methylation level with the RNF185 mRNA expression level in CGGA dataset

At last, the transcription molecular events of RNF185 in glioma were analyzed, and the histone activation marks and DNase-Seq peaks in the promoter region of RNF185 was analyzed in fetal brain, adult brain and glioma cells U87 and U251. As shown in Fig. 7A, strong peak signals were observed in fetal brain, followed by adult brain and glioma cells, suggesting that RNF185 may also under transcriptional repression in glioma. Finally, the DNA methylation level in RNF185 promoter and RNF185 expression was analyzed, with TCGA and CGGA datasets (Fig. 7B, C). The strong negative correlation of methylation with mRNA expression suggests that promoter hypermethylation may play an important role for the decreased expression of RNF185 in glioma samples. What’s more, the gene methylation has significantly relationship with parameter of brain cancer etiology (including Histology type, WHO grade, IDH mutation status and 1p/19q deletion) (Fig. 7D**)**. Especially, gene methylation was remarkably different IDH mutation status (P = 0.032). indicating its clinical significance. Collectively, this study shows that Ring Finger Protein 185 as a novel tumor suppressor in glioma multiforme, which is repressed by promoter hypermethylation and miR-587.

## Discussion

Genomic alterations like IDH1/2 mutation, EGFR amplification and chromosomal 1p/19q deletions have been characterized in glioblastoma pathogenesis, molecular subtyping as well as treatment response [8]. In the past decades, signaling pathway deregulation was shown to be finely tuned by genomic and epigenetic changes, in both transcriptional and post-transcriptional ways [46, 47]. Post-translational modifications, including protein ubiquitination, are well-known for its role in protein metabolism, especially in protein degradation [48]. As a hub link in protein ubiquitination and signaling transduction, the alteration of E3 ubiquitin ligases are emerging as oncogenes and tumor suppressors in cancer, including glioblastoma [14, 49]. Meanwhile, technological advances proposed novel strategies for the un-druggable targets in cancer, such as ubiquitin modifiers and transcription factors [50, 51]. Therefore, revealing the function and molecular mechanism of novel E3 modifiers would deepen our understanding of glioblastoma pathogenesis and provide novel options for its treatment.

CRISPR-Cas9 was first proposed as gene knock-out strategy, and then used for gene activation, inhibition, and other applications [52]. Based on sgRNAs library, CRISPR screening usually include genome wide and targeted screening, such as kinase libraries [53]. Positive and negative selection strategies were also used for uncovering therapeutic targets, drug sensitive and resistant genes [54]. Here, using the hypothesis that cells with certain genes loss of function may gain growth advantage, we acquired 299 differential genes. Considering that the in-vitro experiment may not really reflect the virtual state in clinical glioblastoma samples, we then studied the expression and prognostic significance of differential genes with both TCGA and CGGA datasets. At last, three genes were left: BIRC3, RNF135 and RNF185. Consistent with previous studies, BIRC3 [33–35] and RNF135 [36] were all previously reported as oncogenes in glioma, while we first revealed that RNF185 may play a tumor suppressor role in glioblastoma.

Ring Finger Protein 185 (RNF185) has been shown to regulate cell autophagy [37], innate immune responses [38], osteogenic differentiation [39] and cystic fibrosis [40]. Moreover, RNF185 was proved to promote gastric cancer metastasis [41], while its role in glioma remains to be explored. Interestingly, our screening results in U87 cells showed RNF185 as a tumor suppressor. Further validation with other cell lines U251 and DBTRG also supported the CRISPR screening results. The seemingly contradictory function of RNF185 has also been observed in other E3 ubiquitin ligases in different cancer types. For example, E3 ubiquitin-protein ligase (HUWE1) has been shown to promote cancer progression in lung cancer [55], gastric cancer [56] and multiple myeloma [57], while tumor suppressive functions was also reported in prostate cancer [58] and other cancer types [59]. The converse function of RNF185 may be attributed to its substrates in distinct cancer types.

After validating the RNF185 as a tumor suppressor in glioblastoma, we interrogated the molecular basis for its decreased expression. As a result, we analyzed microRNAs that may induce RNF185 degradation, by bioinformatics prediction, functional studies, and molecular validation assays. MiR-587 was finally proved to repress RNF185 expression, and play an oncogenic role in enhancing cell proliferation, repressing cell apoptosis and induce cell migration and invasion. This is the first time we prove miR-587 as a tumor promoting miRNA in glioblastoma by targeting RNF185. Moreover, we also studied the transcriptional regulators of RNf185 in glioblastoma samples. Histone transactivation marks peaks in fetal, adult brain and glioma cells shows that RNF185 may also under the transcriptional repression, with development and glioblastoma pathogenesis. At last, the DNA methylation levels in RNF185 promoters also supported the above phenomena. Taken together, we conclude that combined transcriptional repression and elevated miR-587 expression may reduce the RNF185 mRNA expression in glioma.

We also recognize that there are still some problems remains to be solved. First, further in-vivo evidence would make the conclusion that RNF185 play a tumor surpassing role in glioblastoma more solid. Secondly, the substrate and exact functional way of RNF185 in glioblastoma remain to be revealed. Thirdly, further studies to confirm the role of the RFP185 as a novel tumor suppressor in a primary culture derived from GBM patients should be performed in the future study. Finally, the strategy of delivering RNF185 for glioblastoma treatment would provide more information for the future clinical application.

## Supplementary Information

**Additional file 1**: **Table S1**. The ubiquitin ligase gene list.

## Data Availability

All relevant data can be acquired by contacting the corresponding author.
